# Levosimendan in Acute and Advanced Heart Failure: an Expert Perspective on Posology and Therapeutic Application

**DOI:** 10.1007/s10557-018-6838-2

**Published:** 2018-11-06

**Authors:** S. Bouchez, F. Fedele, G. Giannakoulas, F. Gustafsson, V.-P. Harjola, K. Karason, M. Kivikko, D. von Lewinski, F. Oliva, Z. Papp, J. Parissis, Piero Pollesello, G. Pölzl, C. Tschöpe

**Affiliations:** 1 Department of Anesthesiology, University Hospital, Ghent, Belgium; 2 Policlinico “Umberto I,” University “La Sapienza”, Rome, Italy; 3 Aristotle University of Thessaloniki, Thessaloniki, Greece; 4 Cardiology, Rigshospitalet, Copenhagen, Denmark; 5 Cardiology Clinic, HUS Meilahti Hospital, Helsinki, Finland; 6 Cardiology, Sahlgrenska University Hospital, Gothenburg, Sweden; 7 Critical Care Proprietary Products Division, Orion Pharma, P.O. Box 65, FIN-02101 Espoo, Finland; 8 Department of Cardiology S7, Jorvi Hospital, Espoo, Finland; 9 Myokardiale Energetik und Metabolismus Research Unit, Medical University, Graz, Austria; 10 Niguarda Ca’Granda Hospital, Milan, Italy; 11 Division of Clinical Physiology, Department of Cardiology, Faculty of Medicine, University of Debrecen, Debrecen, Hungary; 12 Second University Cardiology Clinic, Attiko Teaching Hospital, Athens, Greece; 13 Universitätsklinik für Innere Medizin III Innsbruck, Medizinsche Universität, Innsbruck, Austria; 14 Berlin Center for Regenerative Therapies (BCRT), Campus Virchow Klinikum (CVK), Berlin, Germany

**Keywords:** Inodilators, Inotropes, Acute heart failure, Advanced heart failure, Levosimendan, Renal function

## Abstract

Levosimendan, a calcium sensitizer and potassium channel-opener, is widely appreciated by many specialist heart failure practitioners for its effects on systemic and pulmonary hemodynamics and for the relief of symptoms of acute heart failure. The drug’s impact on mortality in large randomized controlled trials has been inconsistent or inconclusive but, in contrast to conventional inotropes, there have been no indications of worsened survival and some signals of improved heart failure-related quality of life. For this reason, levosimendan has been proposed as a safer inodilator option than traditional agents in settings, such as advanced heart failure. Positive effects of levosimendan on renal function have also been described. At the HEART FAILURE 2018 congress of the Heart Failure Association of the European Society of Cardiology, safe and effective use levosimendan in acute and advanced heart failure was examined in a series of expert tutorials. The proceedings of those tutorials are summarized in this review, with special reference to advanced heart failure and heart failure with concomitant renal dysfunction. Meta-analysis of clinical trials data is supportive of a renal-protective effect of levosimendan, while physiological observations suggest that this effect is exerted at least in part via organ-specific effects that may include selective vasodilation of glomerular afferent arterioles and increased renal blood flow, with no compromise of renal oxygenation. These lines of evidence require further investigation and their clinical significance needs to be evaluated in specifically designed prospective trials.

## Introduction

**Fig. 1:**
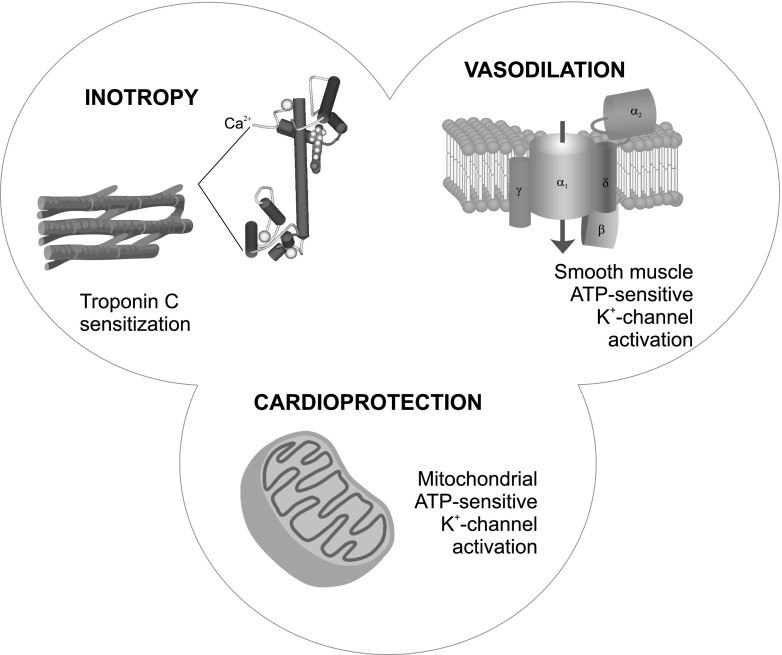
mechanisms of action of levosimendan (data from Papp et al. [1])

The pharmacological effects of levosimendan, an inodilator indicated for the treatment of decompensated heart failure, are exerted via three pathways (Fig. 1) [1]: (1) increased sensitivity of troponin C to calcium in myocardial cells, thereby inducing a cAMP-independent inotropic effect; (2) opening of adenosine triphosphate-sensitive potassium channels (K_ATP_ channels) in the smooth muscle cells of the vasculature, so inducing vasodilation; and (3) activation of K_ATP_ channels in cardiac mitochondria, hence protecting cells against ischemia/reperfusion injury [1]. As it is a calcium sensitizer not a calcium mobilizer [2, 3], levosimendan does not increase myocardial oxygen consumption [4, 5], and prevents myocardial apoptosis and remodeling [6, 7].

In its clinical development program, levosimendan showed beneficial effects on hemodynamic parameters, symptoms, and neurohormones, with a characteristic prolonged pharmacokinetics [8], and an overall positive effect on mortality [9]. Although some pivotal clinical trials failed to show superiority against comparators in terms of survival [10, 11], other regulatory trials [12, 13] as well as the results of the large ALARM-HF registry [14] showed a favorable impact of levosimendan on clinical outcomes, versus either placebo or other inotropes such as dobutamine.

In addition, various lines of clinical investigation have produced indications of a net beneficial impact of levosimendan on renal dysfunction [10, 15–20].

At the 2018 congress of the Heart Failure Association of the European Society of Cardiology (HEART FAILURE 2018, Vienna, Austria, May 27–28), a series of tutorials by experts from nine European countries (Austria, Belgium, Denmark, Finland, Germany, Greece, Hungary, Italy, and Sweden) was delivered examining how to use levosimendan safely and effectively in acute heart failure (AHF) and advanced heart failure (AdHF), including an appraisal of its impact in concomitant renal dysfunction. This review summarizes some of those expert perspectives on the optimized use of levosimendan in these settings.

## Levosimendan in the Clinical Literature

Levosimendan’s current indication for the short-term treatment of acutely decompensated severe chronic heart failure in situations where conventional therapy is not sufficient and where inotropic support is appropriate rests on experience in clinical trials from which a series of salient features was identified.

1. Improved systemic and pulmonary hemodynamics [12, 21, 22] with no significant increase in myocardial oxygen consumption [23, 24]
2. Relief of symptoms [10–12, 21]
3. A theoretically beneficial effect on neurohormone profile [10, 11]
4. Prolonged duration of effect due to the formation of an active metabolite designated OR-1896 [25, 26]
5. Unlike dobutamine, effective also in patients treated with beta-blockers [12, 27]

The safety profile of levosimendan that emerged from these studies was largely reassuring [10, 12, 22], with no impairment of diastolic function [28, 29] or development of tolerance [26]. Common adverse events associated with levosimendan use are hypotension, headache, atrial fibrillation, hypokalemia, and tachycardia [1].

The results of small exploratory clinical trials of levosimendan in AdHF have been summarized by Nieminen et al. [30] and were generally affirmative but inevitably of limited statistical resilience. Two larger studies were subsequently performed and reported: the LEVOREP [31], and LION-HEART [32] clinical trials. As appraised by Pölzl et al. in a consensus opinion paper [33], those studies supported the view that repetitive i.v. use of levosimendan in AdHF patients delivered overall benefit in terms of improved hemodynamics, symptom relief, re-hospitalization rates, biomarkers, and survival but at least one additional suitably-powered clinical trial in AdHF is desirable to set these conclusions on a firm basis of evidence.

Meta-analyses of levosimendan data in various settings, including AHF and AdHF, indicated a trend towards a survival benefit that reached statistical significance in some investigations [34–36] though not all [37, 38]. These discrepancies may have reflected the various selection criteria applied and the precise natures of the study populations. The larger pooled analyses were less likely to identify a robust survival gain and the authors of many of these investigations signaled the need for more extensive prospectively derived data. Of note, however, none of these meta-analyses produced any indication that the use of levosimendan is associated with an increase of mortality, whereas a worsening impact on survival has been reported for other inotropes or inodilators [39, 40].

## Levosimendan: From Guidelines to Clinical Practice

The 2016 ESC guidelines on acute and chronic heart failure assign the same level of evidence and class of recommendation to all inotropes (Class IIb/Evidence level C) but distinguish levosimendan due to its particular mode of action and recommend its use in cases where there is concomitant use of beta-blockers [41]. It is not clear how widely that advice is implemented in real-life clinical practice.

The latest (2018) position statement of the Heart Failure Association of the European Society of Cardiology on Advanced Heart Failure (HFA-ESC) on AdHF [42] comments that “Although patients with chronic heart failure have improved outcomes with implementation of evidence-based therapies, ultimately, they still progress to an advanced stage of the disease.” The authors advise that patients with severe symptoms and reduced exercise capacity, or who endure frequent and repeated hospitalizations, are often refractory to (or cannot tolerate maximal doses of) established therapies and therefore need something in addition to standard-of-care medication.

**Fig. 2:**
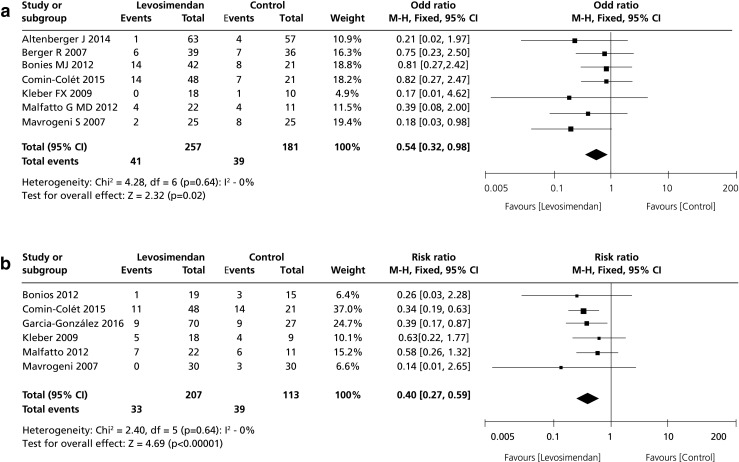
Meta-analyses of the effects of levosimendan on (A) survival (data from Silvetti & Nieminen [35] and (B) re-hospitalization (data from Silvetti et al. [43]) when used repetitively in advanced heart failure

Considered from this perspective levosimendan has attractive features relevant to AdHF and is distinctly different from dobutamine and milrinone. Notably, two meta-analyses associate repeated use of levosimendan in advanced heart failure with improved survival [35] and reduced re-hospitalization [43] (Fig. 2). Given that patients with AdHF may comprise up to 10% of the overall heart failure population [44–46], these benefits may be accessible to substantial numbers of patients. The long duration of action of levosimendan, due to its OR-1896 metabolite [1, 25, 26], is also relevant in this context. The position statement of the HFA-ESC [43] further notes that “…intermittent use of inodilators for long-term symptomatic improvement or palliation has gained popularity, especially use of levosimendan, since the hemodynamic effect may last for >7 days after a 12–24h infusion.” This may be considered as a fair summary of practice in this area.

Guidance and expert consensus [30, 47] advise that levosimendan should ordinarily be administered without a loading bolus (to minimize risk of hypotension). The continuous 24-h infusion of levosimendan should be administered at a rate of 0.05–0.1 μg/kg/min. (Some clinicians start levosimendan infusion at a higher rate, 0.2 μg/kg/min for the first 60 min in order to reach the desired therapeutic effect more rapidly, and then reduce the dose to 0.1 μg/kg/min.) It should be recalled that levosimendan has profound vasodilatory effects; accordingly, it should be administered with caution in patients with low blood pressure. Hypovolemia should be avoided before and during levosimendan treatment (give fluid as needed; reduce intravenous diuretics when necessary) and measures taken to avoid hypokalemia *(*serum/plasma potassium levels should be kept ≥ 4.0 mmol/l during levosimendan infusion).

## Heart Failure and Renal Function: a Role for Levosimendan?

Renal dysfunction is frequently associated with heart failure and is implicated in worse prognosis [48], and further detriment of QoL [49]. Five types of cardiorenal syndrome (CRS) with distinct pathophysiologies and clinical presentations have been described [50].

In patients with heart failure, CRS tends to be encountered as either Type 1 CRS (worsening of renal function during treatment for cardiac decompensation) or Type 2 CRS (reduced glomerular filtration rate [GFR] [< 60 mL/min/1.73 m^2^]): both carry a worse prognosis than heart failure without attendant renal dysfunction. Multiple mechanisms contribute to kidney damage in these forms of CRS, including hypoperfusion, renal venous congestion, interstitial fibrosis, tubular damage, and nephron loss are linked to neurohormonal activation.

The use of inodilators or inotropes may be particularly beneficial in cases of acute CRS in patients with low blood pressure or hypoperfusion in the setting of borderline blood pressure values, and the mechanisms of action of levosimendan identify it as a plausible option in this setting.

**Fig. 3:**
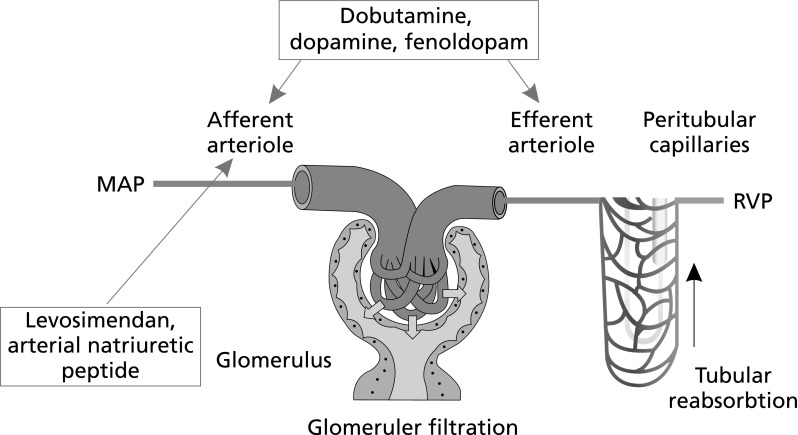
Scheme of the putative selective effect of levosimendan on afferent glomerular arterioles. Levosimendan exerts predominantly a vasodilation of the afferent arterioles [net effect RBF↑, GFR↑], while dopamine and dobutamine vasodilate both afferent and efferent arterioles [net effect RBF↑↑, GFR↔] (from Yilmaz et al. [51])

Evidence for a renal-protective action of levosimendan has been reported from preclinical experiments [51–55]. Improved renal function becomes evident before any increase in cardiac index or left ventricular performance may be detected, suggesting that any renal-protective effect of levosimendan is exerted at least in part via organ-specific effects, including pre-glomerular vasodilation and increased renal artery diameter and renal blood flow, without compromise of renal oxygenation [56, 57]. An interesting hypothesis has been developed to explain those findings: it may be that levosimendan exerts a selective vasodilation on the afferent arterioles of the renal glomeruli thus improving renal filtration both directly and indirectly [51] (Fig. 3).

This finding is consistent with the observation in the Levosimendan Infusion versus Dobutamine (LIDO) trial [12], in which levosimendan treatment was associated with an increase in GFR, whereas treatment with the active comparator, dobutamine, was not even though it increased cardiac index and urine output. The explanation of that disparity offered by the LIDO authors is that the capacity of levosimendan to promote arterial and venous vasodilation through activation of K_ATP_ channels reduces central venous pressure and thus may be beneficial on GFR in some patients.

Further insights into this matter have been provided in recently-reported investigations by Lannemyr, Ricksten, and colleagues in 32 adult patients with chronic heart failure (mean baseline LVEF ~27%) and impaired renal function (mean GFR rate < 80 mL/min per 1.73 m^2^) [58]. As part of an elective cardiac work-up participants were randomly assigned to short-term (75 min) treatments with either levosimendan (loading dose of 12 mcg/kg over 10 min, then infusion at 0.1 μg/kg/min for 65 mins; *n* = 16) or dobutamine (continuous infusion started at 5.0 μg/kg/min for 10 mins then 7.5 μg/kg/min for 65 mins; *n* = 16).

**Fig. 4:**
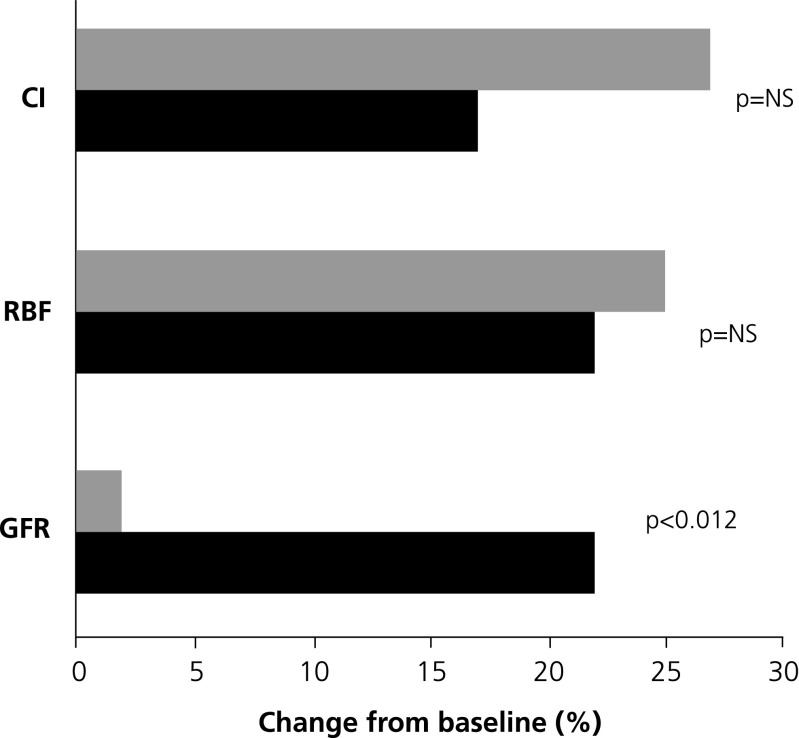
Percentage changes in cardiac index (CI), renal blood flow (RBF), and glomerular filtration rate (GFR) after 75 mins administration of levosimendan or dobutamine. Derived from Lannemyr et al. [58]. (See text for further discussion of dosage and results)

Both treatments were associated with quantitatively and qualitatively very similar alterations in systemic hemodynamic indices, including augmentation of cardiac output and cardiac index, and reductions in systemic vascular resistance index, central venous pressure, and pulmonary capillary wedge pressure. Both treatments also significantly enhanced renal blood flow (levosimendan *P* < 0.05 vs. baseline; dobutamine *P* < 0.001 vs. baseline) but only levosimendan therapy was associated with a significant increase in GFR (*P* < 0.05 vs. baseline: in fact, no alteration in GFR was seen in response to dobutamine, leading to a significant intergroup difference at the end of the treatment phase (*P* = 0.012; Fig. 4). Filtration fraction was stable during levosimendan treatment but fell in response to dobutamine (*P* = 0.045 between groups). The authors of this research conjectured that their data are compatible with the hypothesis that levosimendan acts as a vasodilator on afferent renal arterioles, whereas dobutamine dilates both afferent and efferent vessels. They noted also that as both drugs reduced central venous pressure to a similar extent, this affect could not be adduced as the cause of the different treatments’ impact on GFR. Of note, the > 20% increase in GFR observed in patients treated with levosimendan was not accompanied by renal oxygenation as renal oxygen delivery increased in proportion to the increase in GFR. Lannemyr et al. concede that theirs is a study of short-term drug exposure in a small patient cohort: they suggest, nevertheless, that their data question the assumption that all inotropes that have favorable effects on central and peripheral hemodynamics can be assumed also to exert correspondingly favorable effects on renal perfusion and function, and conjecture that levosimendan "could be the preferred inotropic agent for treatment of the cardiorenal syndrome.”

**Fig. 5:**
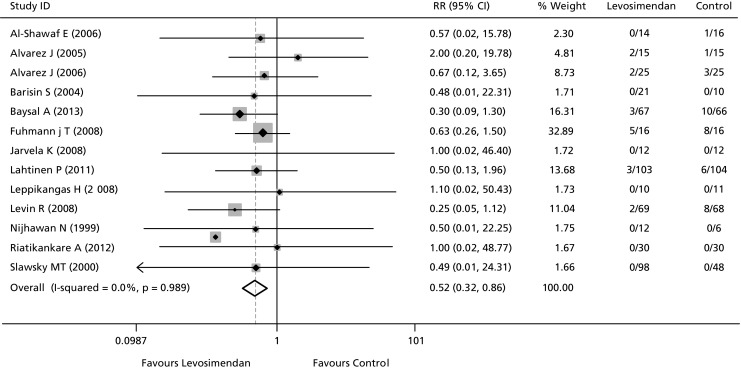
Beneficial impact of levosimendan in critically ill patients with or at risk for acute renal failure: a meta-analysis of randomized clinical trials (data from Bove et al. [60])

Data on the effects of levosimendan on renal function in various clinical situations including cardiac surgery [34, 37, 59] and critical illness [60] have been collated in and the results suggest a renal-protective effect (Fig. 5). Both in these situations, however, and in heart failure, any such effect requires confirmation in specifically designed prospective trials of adequate statistical power.

## Conclusions

Levosimendan’s current indication for the short-term treatment of acutely decompensated severe chronic heart failure is based on its unique pharmacological profile as a myocardial sensitizer and K_ATP_ channel activator and from experience from an extensive clinical trials program.

Renal dysfunction is very common in HF, and a further worsening of kidney function may be expected during hospitalization for AHF. The treatment of cardiorenal syndrome in decompensated HF is challenging due to variable pathophysiology and lack of specifically tailored therapeutic options. Identifying the underlying processes of kidney dysfunction is essential to successful management. Volume status should be checked whenever possible, as well as hypotension and third space fluid accumulation.

Inotropes may be appropriate for short-term management of AHF with renal dysfunction; especially, in low-output states, they may be particularly indicated to avoid renal hypoperfusion. Levosimendan, both in the acute setting and in the repetitive/intermittent context of AdHF, appears to a promising option to improve renal perfusion or to reverse or ameliorate renal dysfunction, but further controlled trials are needed to confirm the status of levosimendan for this purpose.

The posology of levosimendan in AHF and AdHF is a central consideration, especially when treating patients with associated renal dysfunction: dosing of levosimendan should commence without a bolus, to minimize the risk of hypotension. A continuous 24-h infusion of levosimendan should be administered at a rate of 0.05–0.1 μg/kg/min, while maintaining the patient in euvolemic and eukalemic state.
